# A comprehensive representation of the birth-experience: identification and prioritization of birth-specific domains based on a mixed-method design

**DOI:** 10.1186/1471-2393-14-147

**Published:** 2014-04-24

**Authors:** Fania R Gärtner, Liv M Freeman, Marlies E Rijnders, Johanna M Middeldorp, Kitty WM Bloemenkamp, Anne M Stiggelbout, M Elske van den Akker-van Marle

**Affiliations:** 1 Department of Medical Decision Making, Leiden University Medical Centre, P.O.Box 9600, Leiden 2300RC, The Netherlands; 2 Department of Obstetrics, Leiden University Medical Centre, P.O.Box 9600, Leiden 2300RC, The Netherlands; 3 TNO Child Health, P.O.Box 2215, Leiden 2301 CE, The Netherlands

**Keywords:** Labor and birth experience, Utility measure, Patient-reported outcomes, Questionnaire, Cost-effectiveness

## Abstract

**Background:**

In obstetrics, effectiveness and cost-effectiveness studies often present several specific outcomes with likely contradicting results and may not reflect what is important for women. A birth-specific outcome measure that combines the core domains into one utility score would solve this problem. The aim of this study was to investigate which domains are most relevant for women’s overall experience of labor and birth and should be included in such a measure.

**Methods:**

A sequential mixed-method design with three steps was applied. First, the domains were identified by literature review and online focus groups consisting of pregnant women, women who recently gave birth, and their partners. Second, in a prioritizing task, women who recently gave birth and professionals (midwives, gynecologists, and researchers) selected and ranked their top seven domains. Third, the domains that were most frequently selected and had the highest ranking scores determined the basis for a consensus discussion with experts, whereby the definitive list of domains was formed.

**Results:**

In the first step, 34 birth-specific domains were identified, which cover domains regarding the caregivers, intrapersonal aspects of the mother, partner support, and contextual and medical aspects of birth. Based on the prioritizing task results (step 2) of 96 women and 89 professionals, this list was reduced to 14 most relevant domains. In a consensus discussion, the final seven domains were selected by combining several of the 14 remaining domains and giving priority to the domains indicated to be relevant by mothers. The seven definite domains were: 1) availability of competent health professionals; 2) health professionals’ support; 3) provision of information; 4) health professionals’ response to needs and requests; 5) feelings of safety; 6) worries about the child’s health; and 7) experienced duration until the first contact with the child.

**Conclusions:**

The experienced availability and quality of received care, concerns about safety and the baby’s health, and first contact with the baby are regarded as key aspects for a mother’s overall birth experience. Therefore, these domains are considered to be the most crucial for inclusion in a birth-specific outcome measure.

## Background

In medical research, innovative health care interventions are constantly evaluated and compared with routine care. The evidence derived from these effectiveness and cost-effectiveness aims to provide input for medical decision making for patient care [1]. The preferred outcome measure for effectiveness is the Quality Adjusted Life Year (QALY), which is assessed using classification systems, such as the EQ-5D and SF 6D [2,3]. In obstetric studies that compare different interventions during the course labor and birth, using these measures may be less appropriate, especially in situations in which only the course of labor and birth is affected and no long-term differences in health outcomes of mother and child are expected. Because of the relatively short duration of labor, the differences between interventions that affect only the course of labor and birth will not be reflected in QALY estimates. The use of an obstetric-related specific measure that concentrates on the course of labor and birth is, therefore, more informative in these situations. In the absence of such a measure, many (cost) effectiveness studies in obstetrics focus on specific aspects of labor, such as pain [4,5], patient satisfaction [6], labor duration [7], or anxiety [8,9].

Currently, these indicators are typically considered separately; one indicator is considered as a primary outcome measure and several others are considered as secondary outcome measures. These different outcomes may be contradictory, e.g., the amount of pain perceived during labor might be inconsistent with the quality of the care received during labor as experienced by the mother. Therefore, a composite outcome measure that reflects the core domains of the labor and birth based on the women’s experience and combines these domains into one utility score would solve the problem of separate outcome presentation [10]. A preference-based utility measure with a weighting function is regarded to be most suitable for achieving this aim, because the levels of the domains might contribute differentially to the overall experience. For example, support by health care professionals and the patient’s role in decision making might be of greater influence on the labor and birth experience than mode of delivery [11,12].

In existing multidimensional self-report questionnaires for obstetric outcomes, the differences in the subscales’ extent of contribution to the overall experience of labor and birth are not considered [6,13-15]. Because of the large number of items, calculating such weighting functions for the utility score of the existing questionnaires is beyond the means of current analyzing methods, such as discrete choice analysis [16]. A number of six or seven domains is regarded to be the maximum for cognitively coping [17,18].

Before a new composite outcome measure can be developed, it is necessary to investigate which domains of labor and birth are most relevant for women’s overall experience of labor and birth. Therefore, as a first step in this development process in the present study, we reported results for the following research questions: First, what are the birth-specific domains that play a role in women’s experience of labor and birth? Second, what are the birth-specific domains (seven at most) regarded to be most relevant for women’s experience of the process of labor and birth?

## Methods

The present study holds a mixed-methods design and consists of three steps [19]. In the first qualitative step, which was inductive in nature, we identified birth-specific domains (from now on, this term is referred to as “domains”) by conducting a scoping literature review, online focus groups with the target group, and an assessment of expert opinions (midwives and obstetricians). In the second step, we used quantitative methods to appraise the relevance of the domains for the overall experience of labor and birth. In this deductive step, an online prioritizing task was presented to women who recently gave birth and to professionals and researchers in obstetrics. The third step aimed at reducing the most important domains into a definite list of seven domains; to achieve this aim, an expert meeting with consensus discussion was held. The set-up and analyses followed a sequential design, in which step two builds on the results of the previous step. The last step finalizes this process with an expert discussion [19].

The Medical Ethics Committee of the Leiden University Medical Centre approved this study.

### Step 1: Identification of birth-specific domains

#### ***Scoping review***

##### 

**Literature search** The aim of the scoping review search was to investigate existing literature concerning birth experiences to gain a comprehensive overview of likely domains. The term “scoping review” refers to an approach in which a rapid search is conducted to map evidence in a high-volume research area. Using this method, the depth and broadness of evidence coverage vary depending on the search aim. Because of the explorative nature of our research questions, the scope of our review was broad instead of a review of in-depth evidence [20,21].

Three electronic databases (PubMed, Web of Science, and PsycINFO) were systematically searched for peer-reviewed articles in March 2011. For the search strategy, three search groups were combined with the Boolean operator AND; first, a group of search terms for obstetrics, labor, and birth; second, a group of search terms for outcomes and experiences; and third, search terms for broad study outcomes, such as “outcome and process assessment” or “questionnaire”. For each of the three groups of search terms, index terms specific for each database (such as MESH terms in PubMed) were combined with free-text words for searching in the title or in the title and abstract. The complete search strategy is presented in the Additional file 1. The selected scientific articles were supplemented with key articles identified by the research team.

##### 

**Selection of studies** All retrieved articles were evaluated for eligibility based on the title and abstract. If this evaluation did not provide adequate certainty about inclusion, the article was evaluated based on its full-text. Articles were eligible for inclusion in the study if the articles either

a. studied one or more domains in a specific population or compared the domain(s) between different populations,

b. identified one or more domains,

c. used one or more domains as outcome measure,

d. studied the relationship between different domains, or

e. evaluated or developed a questionnaire that measures one or more domains.

As a domain, we regarded all experiences or emotions of the mother related to the process of labor and birth or to the period shortly after birth (maximum of one week). Excluded were articles that present systematic reviews, except for reviews that aim to inventory domains on birth experience. Additionally, case reports were excluded because we included individual experiences, to a large extent, by the focus group data. Also excluded were articles that studied the outcomes of specific obstetric interventions, which detracted from generalizability (e.g., pain of applying a labor induction method or satisfaction with specific aspects of a birth plan), and articles that were not written in English, German, or Dutch.

##### 

**Data extraction and analyses of the literature search** For each included article, the key terms describing the domains that are investigated in the article were inventoried. Depending on the type of study, this inventory could be variables, for example, that determine group differences, outcome variables used, results from qualitative data, or questionnaire subscale. A data-driven thematic analysis of the data extracted from the included articles was performed [22]. First, open coding was applied to the extracted data by one researcher (F.G.). Second, the various codes were categorized into themes that shared common aspects of labor and birth. The categorization was performed by one researcher (F.G.) and checked by a second researcher (E.v.d.A.-v.M.). In case of disagreement, category allocation was discussed between the two researchers until consensus was reached.

#### ***Focus group interviews and expert opinions***

##### 

**Recruitment of focus groups participants and experts** Three online focus groups were convened, one for pregnant women, one for women who recently gave birth, and one for partners of the latter group. The participants were recruited between September and November 2011. The participants had to be at least 18 years, conversant in Dutch, and meet one of the following criteria: be in the third trimester of pregnancy, have recently (within 12 months) given birth, or be a partner of the latter group of women. The participants were recruited from the Department of Obstetrics and Gynecology of a Dutch academic medical center, through primary-care midwifery practices, and through calls on websites for this target population. The focus group data were supplemented with keywords that were presented by midwives and obstetricians who were part of the research team network.

##### 

**Procedure of focus groups and experts data gathering** After providing their written informed consents, the participants received their log-in data for the online forum and registered using a self-chosen username. The respondents received an information leaflet that provided the aim of the study, the date, and the time of the focus groups. The respondents were instructed to log in at least once, but they were encouraged to log in more often and to react to each other’s comments. The online focus groups were structured according to two questions that were slightly adapted for the three different groups of respondents. First, the participants were asked to introduce themselves and to provide key aspects concerning labor and birth. After 24 hours, the second question was posed to the forum, which asked participants to explain which domains were predominant for their (or their partners’) overall experience of labor and birth. The moderator (E.v.d.A.-v.M.) occasionally summarized the answers and encouraged the participants to clarify their keywords and to react to each other’s answers.

The professionals’ opinions were assessed by means of an open-ended question: “*What are the most important aspects concerning mother and child that should be included in a composite outcome measure on birth experiences?*” For the obstetricians, this question was part of a larger questionnaire from another study that encompassed 90 hospitals in the Netherlands, accompanied with the request that the head of the gynecology department or a colleague who specialized in obstetrics complete the form. The midwives received this question by e-mail.

Professionals’ opinions were not gathered during focus groups because the professionals were very familiar with the topic and they are accustomed to stating their opinions; therefore, it was considered less necessary to stimulate reflection during group discussion, as is done in focus groups.

##### 

**Analysis of focus group data and expert opinions** The analysis of the focus group and expert opinion data followed a purpose-driven approach that aimed to distinguish as many different aspects of labor and birth as possible. First, the transcripts of each focus group were open-coded. Second, axial coding was performed. Within this process of re-rereading, the codes were refined, reduced, and related to each other. Third, the obtained codes were categorized into themes that covered related aspects of labor and birth, which resulted in the list of the domains [23,24]. One researcher (F.G.) performed the data coding; subsequently, a second researcher (E.v.d.A.-v.M.) studied the coded data in each focus group and the expert’s key terms; any inconsistencies were discussed and solved in a consensus-discussion between the two researchers. All of the qualitative analyses were performed using the software ATLAS.ti (Scientific Software Development GmbH, Berlin, Germany) [25].

### Data synthesis of literature and qualitative data

The domains derived from the literature search and the focus group data were synthesized into one list of domains. For each domain, a definition was formulated, discussed, and rephrased in the research team until a consensus was reached.

### Step 2: Prioritizing task

#### ***Participants of the prioritizing task***

To be included in the sample of women who recently gave birth, women had to have given birth between October 2012 and February 2013 and be conversant in Dutch. There was no restriction on any obstetric characteristic because we aimed to achieve diversity in the sample. Women were recruited via flyers at obstetric outpatient clinics and primary-care midwifery practices, as well as calls on the intranet and the official website of a Dutch academic medical center, information on websites for pregnant women, and Twitter and Facebook accounts of midwives.

The obstetricians and midwives were recruited via the personal network of the research team members. The link to the online questionnaire was e-mailed to 86 midwives or primary-care midwifery practices and to 56 obstetricians, accompanied with the request to complete the questionnaire and to forward the e-mail to other professionals who might be interested. Twenty-one researchers working in the field of obstetrics in the Netherlands were recruited via a personal e-mail invitation.

#### ***Procedure of the prioritizing task***

During January and February 2013, the online questionnaire was distributed by e-mail to the participants. In this online questionnaire, 34 identified domains with their definitions were presented. The appraisal of the relevance of the domains contained three parts. First, to become familiar with the domains and their definition, the domains were presented singly to the respondent. The respondent was asked to rate the perceived relevance for each domain using a 5-point scale (0 = *not relevant*, 1 = *slightly relevant*, 2 = *relevant*, 3 = *very relevant*, 4 = *extremely relevant*). This relevance score was dichotomized into “non-substantial” (scores 0, 1, and 2) and “substantial” (scores 3 and 4). We chose to not include the middle category “relevant” in the categorization of “substantial” domains because based on the methods we used for generating this list of 34 domains, we expected all of the domains to be relevant. However, to distinguish within this list of relevant domains, we believed that the domains should be of extra importance.

Subsequently, the seven most relevant domains had to be selected from the total list of domains by asking them to perform the following: “*Please choose seven aspects from the list of 34 aspects of labor and birth that you regard as most important for the overall experience of labor and birth*”. Finally, the seven selected domains had to be ranked from the most relevant to the least relevant.

The online questionnaire for women who recently gave birth included questions on obstetrical background (number of pregnancies, miscarriages, and parity), facts about the most recent birth (place of birth, method of birth, single versus multiple pregnancy, use of pain relief during labor, complications during labor, maternal and neonatal morbidity), and demographic and work characteristics (age, marital status, ethnical background, level of education, and employment status status). For the professionals, additional questions on gender, profession, and years of work experience were asked to gain insight into the characteristics of the sample.

#### ***Analyses of the prioritizing task***

Based on the data of the prioritizing task, a list of the most important domains was identified. For this list, the seven domains with the highest proportion of the top seven selection (part 2) and the seven domains with the highest mean ranking score were included (part 3). This analysis was done separately for the two datasets, i.e., the sample of women who recently gave birth and the sample of professionals.

All statistical analyses were performed using IBM SPSS 20.0 (Armony, NY, IBM) [26].

### Step 3: Expert meeting with consensus discussion

In the last step, an expert meeting with a consensus discussion was held to reduce the list of most important domains, derived in step two, to a maximum of seven domains. To achieve this aim, the domains were combined or reduced. All of the decisions made by the experts had to guarantee that the final domains fulfilled the following three requirements:

1. The domains are clearly distinguished and independent of each other.

2. Each domain must be measurable by self-reports of the mother.

3. Each domain must be applicable to all types of deliveries, regardless of place and type of labor, e.g., home versus outpatient clinic versus hospital, and vaginal versus caesarean section.

Furthermore, we decided that the results of the sample of women who recently gave birth were leading for the decisions made during the reduction process. The reason for this choice was that the instrument to be developed aimed to measure the experience of the women who gave birth and thus predominantly should present the domains they regard as important. However, overall, the final list of domains must be supported by the data of the professionals.

For the expert meeting, we invited midwives, obstetricians, and researchers in the fields of obstetrics, health economics, or clinimetrics. The experts were recruited from the researchers’ personal network and they were members of the research team.

## Results

### Step 1: Results of the domain identification

#### ***Literature search***

A total of 2,060 titles were identified by the literature search: 959 in Pubmed, 828 in Web of Science, and 273 in PsycINFO. After eliminating 211 duplicates and adding five relevant articles by the research team, 1854 articles were reviewed for inclusion, of which 170 articles met the inclusion criteria. Of these 170 included articles, 70 fell into inclusion category a (studied one or more birth-specific domains in a specific population or compared the birth-specific domain(s) between different populations); 11 fell into category b (identified one or more birth-specific domains), 15 fell into category c (used one or more birth-specific domains as outcome measure), 43 fell into category d (studied the relationship between different birth-specific domains), and 31 fell into category e (evaluated or developed a questionnaire with one or more birth-specific domains as its construct). We regarded the data to be saturated because each domain identified in the literature was derived from at least two papers, but in the majority of cases by more than four papers. Because of the extent of the list of included articles, the references are not included in this paper but can be provided from the corresponding author.

#### ***Focus groups and expert opinions***

For the online focus groups, 34 participants registered, of whom 29 participants in fact participated: nine pregnant women, 13 women who recently gave birth, and seven partners. Their mean age was 32.4 years (SD = 3.6). Fifty-five obstetricians and midwives responded to the call to name aspects of labor and birth that are crucial to the evaluation by women. In total, 55 professionals provided key terms and their opinions concerning the most important aspects of birth.

#### ***Identified birth-specific domains***

In total, 34 domains were identified (Table 1). Of these domains, 28 overlapped between the literature search and focus group results, four domains were derived solely from the literature data (*transfer between medical staff*, *freedom of movement during labor*, *duration of labor and* birth, *compliance with expectations*) and two domains were derived solely from the focus group and expert data (*support of partner by health professionals, active involvement of partner by health professionals*).

**Table 1:** Results of the prioritizing task for two samples: women who recently gave birth and professionals

		**Women who gave birth (N = 96)**						**Professionals (N = 89)**					
		**Step 1 (relevance score)**		**Step 2 (selection of top 7)**		**Step 3 (ranking of top 7)**^**†**^		**Step 1 (relevance score)**		**Step 2 (selection of top 7)**		**Step 3 (ranking of top 7)**^**†**^	
**Domains**		**n**	**(%)**	**n**	**(%)**	**m**	**(SD)**	**n**	**(%)**	**n**	**(%)**	**m**	**(SD)**
* 1	**Competence of health professionals**	93	(97)	72	**(75)**	**5.46**	(1.4)	75	(84)	52	**(58)**	**5.00**	(1.9)
* 2	**Treatment by health professionals**	90	(94)	24	(25)	3.29	(1.6)	81	(91)	38	**(43)**	4.16	(2.0)
3	Support of partner by health professionals	41	(43)	4	(4)	2.75	(2.1)	47	(53)	0	(0)	-	-
4	Active involvement of partner by health professionals	53	(55)	4	(4)	1.75	(0.5)	43	(48)	5	(6)	4.00	(1.4)
* 5	**Provision of clear information by health professionals**	87	(91)	45	**(47)**	3.40	(1.7)	85	(96)	35	**(39)**	3.94	(1.7)
6	Involvement of parents in decision making	75	(78)	22	(23)	3.55	(1.8)	75	(84)	34	(38)	3.76	(1.9)
7	Health professionals consider wishes of parents	71	(74)	15	(16)	3.00	(1.5)	65	(73)	24	(27)	3.21	(1.8)
8	Health professionals consider specifics concerning the pregnancy or previous labor experiences of the women	70	(73)	15	(16)	2.87	(2.2)	80	(90)	10	(11)	3.20	(1.5)
* 9	**Support of the mother by health professionals**	84	(88)	26	(27)	**4.08**	(1.7)	79	(89)	15	(17)	3.73	(1.9)
*10	**Availability of health professionals when needed by the mother**	77	(80)	19	(20)	**4.16**	(1.8)	72	(81)	25	(28)	3.60	(1.6)
11	Feeling at ease with the health professionals	65	(68)	15	(16)	2.60	(1.7)	57	(64)	9	(10)	3.00	(1.7)
*12	**Transfer between medical staff**	75	(78)	19	(20)	3.11	(2.0)	76	(85)	37	**(42)**	3.14	(1.9)
*13	**Support of the mother by her partner**	74	(77)	21	(22)	3.62	(1.7)	39	(44)	4	(5)	**4.75**	(1.5)
14	Preparation of the mother for labor	41	(43)	5	(5)	4.00	(2.4)	31	(35)	9	(10)	3.67	(2.6)
*15	**Self-confidence of the mother**	64	(67)	14	(15)	**4.71**	(2.0)	66	(74)	26	(29)	**5.15**	(1.8)
16	Feelings of control of the mother over the situation	50	(52)	15	(16)	3.93	(1.6)	54	(61)	23	(26)	4.13	(2.0)
*17	**Trust of the mother in a good course of labor**	78	(81)	28	**(29)**	2.71	(1.5)	67	(75)	22	(25)	**4.55**	(1.7)
18	Fear of the mother during labor	47	(49)	14	(15)	3.00	(2.3)	71	(80)	25	(28)	4.28	(1.6)
*19	**Stress of the mother during labor**	42	(44)	3	(3)	2.00	(1.7)	42	(47)	4	(5)	**4.50**	(1.3)
*20	**Feelings of safety of the mother during labor**	70	(73)	38	**(40)**	3.58	(1.7)	73	(82)	50	**(56)**	4.42	(1.9)
21	Place of birth	30	(31)	3	(3)	3.33	(2.3)	36	(40)	4	(5)	3.50	(1.9)
22	Transfer during labor	17	(18)	1	(1)	1.00	(-)	20	(23)	2	(2)	1.00	(0.0)
23	Atmosphere at the place of labor	38	(40)	10	(10)	1.80	(1.8)	52	(58)	9	(10)	2.56	(1.6)
24	Method of birth	60	(63)	7	(7)	3.29	(1.3)	50	(56)	9	(10)	3.11	(2.1)
25	Medical interventions during labor	46	(48)	16	(17)	2.75	(1.7)	45	(51)	9	(10)	2.56	(1.4)
26	Freedom of movement during labor	34	(35)	6	(6)	3.00	(2.2)	53	(60)	13	(15)	2.46	(1.5)
27	Pain perception	58	(60)	9	(9)	3.44	(1.6)	57	(64)	12	(14)	3.00	(1.8)
28	Pain relief	52	(54)	23	(24)	3.13	(1.9)	47	(53)	7	(8)	1.43	(0.8)
29	Physical strain	34	(35)	1	(1)	4.00	(-)	28	(32)	1	(1)	2.00	-
*30	**Worries concerning the child’s health during and right after labor**	96	(100)	79	**(82)**	**5.95**	(1.7)	79	(89)	41	**(46)**	**5.22**	(1.7)
*31	**Worries about the health of the mother during and right after labor**	78	(81)	40	**(42)**	**4.75**	(1.7)	65	(73)	28	**(32)**	**5.75**	(1.8)
32	Duration of labor	31	(32)	4	(4)	4.00	(2.9)	19	(21)	1	(1)	4.00	-
*33	**Duration until first contact with the child**	82	(85)	51	**(53)**	**4.16**	(1.6)	73	(82)	27	(30)	3.26	(2.2)
34	Comply with expectations	27	(28)	4	4	1.50	(0.6)	36	(40)	13	(15)	2.00	(1.7)

^†^Ranking scores range from 1-7, with 7 = most relevant and 1 = least relevant.

**Bold text**: Step 2: seven domains with the highest proportion being selected in the top 7; step 3: seven domains with the highest mean ranking score.

*14 domains selected as the most relevant based on both samples.

The list of domains comprises the domains regarding caregivers (such as *competence of caregivers* and *treatment by caregivers*), the role of the partner (such as *support of the mother by her partner*), more intrapersonal domains of the mother on the experience of labor (such as *fear of the mother during labor* and *feelings of control of the mother over the situation*), domains regarding facts on the environment and method of the birth and interventions (such as *place of birth* and *pain relief*), and other domains (such as *first contact with the child)*.

### Step 2: Results of the prioritizing task

In total, 122 women subscribed for the prioritizing task; 96 women completed the questionnaire (79% response rate) (Table 2). On average, the women were 33 years old. The majority of women were married or living together, Dutch, had finished a higher level of education, or university, and were employed or self-employed. Approximately 54% were primipara; on average, birth was 8.8 weeks ago; and the duration of pregnancy was 39.7 weeks. Twenty-four percent of the deliveries were led by a primary-care midwife, and 11.5% of the total sample gave birth at home. Of all respondents, 24% reported complications concerning the child and 29% reported complications concerning themselves.

**Table 2:** Participant characteristics of the prioritizing task

**Sample of women who recently gave birth (n = 96)**		
***Demographic characteristics***		
*Age in years (mean, (SD))*	32.7	(3.9)
*Marital status (n, (%))*		
Married/living together with partner	95	(99)
In a relationship (not living together)	1	(1)
*Ethnical background (n, (%))*		
Dutch	82	(86.3)
Second-generation immigrants	6	(6.3)
First-generation immigrants	7	(7.4)
*Highest level of education (n, (%))*		
Primary school	0	(0)
Secondary school	5	(5.2)
Vocational education	9	(9.4)
Higher level education	32	(33.3)
University	7	(52.1)
*Employment status (n, (%))*		
Employed or self-employed	87	(90.6)
Housekeeping	3	(3.1)
Student	1	(1)
Volunteer work	1	(1)
Unemployed	4	(4.2)
***Obstetrical history and characteristics***		
*History of terminated pregnancy, miscarriage, or extra-uterine pregnancy (n, (%))*	4	(0.4)
*Parity (n, (%))*		
Primipara	52	(54.2)
*Age of baby at time of filling out questionnaire (in weeks) (mean, (SD))*	8.8	(5.3)
*Gestational age of newborn (in weeks) (mean, (SD))*	39.7	(1.64)
*Type of pregnancy (n, (%))*		
Singleton	94	(97.9)
*Place of birth (n, (%))*		
Home led by midwife from primary-care practice	11	(11.5)
Birth center led by midwife from primary-care practice	1	(1)
Hospital led by midwife from primary-care practice	11	(11.5)
Hospital led by secondary-care midwife	24	(25)
Hospital led by obstetrician	49	(51)
*Onset of labor (n, (%))*		
Spontaneous	67	(69.8)
Induced	29	(30.2)
*Mode of birth (n, (%))*		
Vaginal	65	(67.7)
Instrumental vaginal (e.g., ventouse or forceps)	12	(12.5)
Planned caesarean section	2	(2.1)
Emergency caesarean section	17	(17.7)
*Use of pain medication during labor (n, (%))*		
No	50	(52.1)
Yes, epidural	29	(30.2)
Yes, other than epidural	17	(17.7)
*Labor complications concerning the child (n, (%))*	23	(24)
*Labor complications concerning the mother (n, (%))*	28	(29.1)
**Sample of professionals (n = 89)**		
*Age (mean, (SD))*	39.5	(10.2)
*Gender*		
Female *(n, (%))*	85	(95.5)
*Occupation (several answers possible)*		
Midwife in practice	41	(46.1)
Midwife in hospital	9	(10.1)
Obstetrician in training	9	(10.1)
Obstetrician	7	(7.9)
Researcher	14	(15.7)
Teacher	3	(3.4)
Other	5	(5.6)
*Work experience (in years) (mean, (SD))*	13.1	(9.5)

Regarding the obstetric characteristics, the composition of this sample primarily corresponds with the data of The Netherlands Perinatal Registry of 2010. The primary differences are that our sample includes fewer immigrants (13.7% versus 22.7% in The Netherlands in 2010), slightly more primipara (54.2% versus 47.5%), slightly fewer home births (11.5% versus 17.1%), fewer planned caesarean sections (2.1% versus 7.6%), but more emergency caesarean sections (17.7% versus 9.2%), and more pain medication by epidural anesthesia (30.2% versus 15.9%). The mean age when giving birth was similar, i.e., 32.7 years in our sample and 32.1 years in The Netherlands in 2010 [27].

In the group of professionals, 89 persons completed the questionnaire. On average, they were 39.5 years old and had a mean work experience of 13 years. In this sample, 96% was female and 56% were midwives, 23% obstetricians or residents in obstetrics, and the remaining respondents were researchers, teachers, or had other professions.

In the prioritizing task, 14 domains were selected as most relevant (Table 1). In Table 1, the 14 selected domains are marked with an asterisk [*] and bold. Comparing the most important domains of the two samples, seven domains overlapped. Women appeared to regard domains concerning support and availability of health professionals as more relevant than their own emotions (*stress during labor)* and the role of their partner. The professionals regarded domains concerning stress of the mothers and the support of the mother by the partner as more important than their own role (*support and availability of health professionals*). Furthermore, it is notable that in both samples, the domains concerning the course of labor (such as *pain perception* and *duration of labor*) and medical and environmental aspects (such as *method of birth, medical interventions during labor, place of birth,* and *atmosphere at the place of labor and birth*) are not represented in the total list of most important domains.

### Step 3: Results of the expert meeting

In the consensus discussion, the final seven domains were selected by combining several of the 14 remaining domains and giving priority to the domains indicated to be relevant by mothers. The seven definitive domains derived from the expert meeting were: 1) availability of competent health professionals; 2) support by health professionals; 3) provision of clear information by health professionals; 4) health professionals’ response to the mother’s needs and requests; 5) feelings of safety; 6) worries about the child’s health state; and 7) experienced duration until the first contact with the child.

The following decisions and rationale underlie this final list:

• Domains 20 (*feelings of safety*), 30 (*worries about the health of the child*), and 33 (duration until the first contact with the child) were included in the definite list of domains because they fulfill all requirements.

• Domains 1 (*competence of health professionals*) and 10 (*availability of health professionals*) were combined because the availability of caregivers is regarded to be only valuable in combination with their competence; for example, the presence of midwives or doctors in training might not always be perceived as positive by women in labor.

• Domains 2 (*treatment by health professionals*) and 9 (*support of the mother by health professionals*) were combined because these were regarded to be not independent of each other.

• Domain 12 (*transfer between medical staff*) was deleted from the list because a subjective measurement by self-report of the mother was regarded to be not likely.

• Domains 13 (*support of the mother by her partner*), 17 (*trust of the mother in a good course of labor*), and 19 (*stress of the mother during labor*) were removed by the experts because they did not stand out in the prioritizing results of the sample of women who recently gave birth.

• Domain 15 (*self-confidence of the mother*) was removed from the final list because only a small group of women (15%) chose this domain in their top seven; therefore, the experts regarded this domain to be inferior to the other domains.

• Domain 31 (*worries about the health of the mother*) was deleted because it was regarded to overlap with domain 20 (*feelings of safety*).

• Domains 6, 7, and 8 on personalized care (*involvement of parents in decision making, health professionals consider wishes of parents, health professionals consider specifics concerning the pregnancy or previous labor experiences of the women*) were combined into one extra domain concerning the caregivers’ response to the mothers’ needs and requests because it is regarded to be a crucial component of personalized care.

## Discussion

The three research steps of this study lead to the identification, prioritization, and selection of domains that are regarded to be most relevant for women’s overall experience of labor and birth. The seven definitive domains are: 1) availability of competent health professionals; 2) health professionals’ support; 3) provision of clear information by health professionals; 4) health professionals’ response to the mother’s needs and requests; 5) feelings of safety; 6) worries concerning the child’s health state; and 7) experienced duration until the first contact with the child.

### Interpretation of results and comparison with previous studies

Interestingly, the results of the prioritizing task indicate that domains regarding the health professionals’ behavior and attitude, as well as domains regarding the mothers’ feelings of confidence and safety prevail over domains regarding the course of labor and the medical and the environmental aspects of the birth. These findings are consistent with the results of a systematic review of predictors for childbirth experience [12]. Hodnett and colleagues concluded that intrapartum medical intervention as well as the model of care and birth environment, are important for the childbirth experience; however, these domains were considered to be less important than the influence of the health professionals’ attitude and behavior. These findings led us to assume that aspects that cannot necessarily be influenced by the mother or health professionals are less important for the overall experience than aspects that health professionals and mothers have influence on. Furthermore, the findings let assume that aspects of the birth that under circumstances jeopardize a safe birth, such as the method of birth, in cases where a vaginal birth is not regarded to be safe, are less important for the overall experience as well.

Another conspicuous result of the prioritizing task is that the domain *involvement of parents in decision making* (domain 6) did not dominate in the top 7 selection, especially in the sample of women who recently gave birth. In contrast, in the results of the review of Hodnett and colleagues and also in various birth experience scales, involvement in decision making plays an important role in the evaluation of the birth experience [6,11-13,28,29]. The low evaluation of this domain might be explained by the fact that in the list of 34 domains, i.e., a number of domains were related to care that is attuned to the individual needs and wishes, i.e., *involvement of parents in decision making* (domain 6), *provision of clear information* (domain 5), *considering wishes* (domain 7), and *considering specifics on the pregnancy or previous labor experience* (domain 8). In the prioritizing task, domains that are similar might have competed with each other. Respondents might have nominated seven different and independent domains, rather than domains that overlapped in their meaning, to obtain a top 7 selection that represents as many different aspects as possible. Thus, the domains on personalized care might have oppressed each other, and none of them clearly stood out in the selection and ranking scores, except for the *provision of clear information* (domain 5). These considerations were discussed in the expert meeting. The group concluded that the following two key components of shared decision making should be reflected in the final list of domains: 1) informing the patient and 2) personalizing care based on the individual needs and wishes [30,31]. Therefore, the expert group decided to combine the original domains on personalization of care into one domain and include it in the final list of seven domains. This final list is formulated as “the health professionals’ response to the mothers’ needs and requests”. This domain aims to be receptive to the mothers’ unfulfilled needs, e.g., negative experiences concerning the options for pain medication.

### Methodological considerations

The bottom-up approach to inventory the domains was a strength in this study. A mixed-methods approach was used to derive a comprehensive list of domains, and both women’s and professionals’ experiences of labor and birth strongly contributed to the result.

A possible concern regarding the validity of the results for the prioritizing task arises from the composition of our sample of women who recently gave birth. A selection bias might have occurred because women with a higher educational degree were overrepresented, as well as respondents of Dutch origin. In future studies, and especially in the determination of utility values for the seven definite selected domains, it is important to improve the participation of lower educated women and immigrant women.

Furthermore, the validity of our results outside The Netherlands should be discussed. Hodnett and colleagues concluded that in their review, the results of the experience of childbirth are consistent regardless of the country [12]. The obstetric care system in The Netherlands is unique. Here, women who expect a physiological birth are directed to primary-care midwives and the rates of home-birth and midwife-led hospital births are high compared to the rates in other European countries [27]. However, interestingly, domains that might be related to the Dutch culture in obstetric care were not highly selected by women or professionals, such as place of birth and medical interventions during labor and birth, and thus are not included in the definitive list of the seven domains. Furthermore, the final list of seven domains had to be applicable to all kinds of deliveries, regardless of the place and mode of birth; consequently, the domains are broadly applicable. In conclusion, the validity of our results outside The Netherlands is expected to be good but should be further explored.

Regarding the generalizability within the Dutch setting, we note that our results derived from a single setting study. The replication of the study in the same cultural context, but with a larger sample size, would strengthen the validity of the results.

### Implications for future research and practice

In the present study, we investigated which domains of labor and birth are regarded to be most relevant for women’s overall experience of labor and birth and we derived a final selection of seven domains. As explained in the introduction of this study, a composite outcome measure is needed for the cost-effectiveness studies in obstetrics [10]. The results of the present study provide a basis for the development of a composite outcome measure. To achieve this aim, suitable items and response categories need to be formulated based on the seven domains selected here, and a study on the measurement characteristics of the new utility measure should be performed. Furthermore, for such questionnaires, a weighting function should be estimated by means of which a single utility score can be calculated, that ranges from 0 to 1.

In addition to using the results for developing a utility measure, the results of this study are of relevance for obstetric professionals and policy makers. The prioritizing results showed that there are several discrepancies in what women who recently gave birth and professionals regard to be important for the overall evaluation of the experience of labor and birth. Based on the results of part two of our study, it might be concluded that women regard the attendance and support of health professionals to be more important than what the professionals think it is for the evaluation of the labor. This fact underscores the importance of the psychosocial portion of the care provided in obstetrics. In future research, these incongruences need to be further studied, as well as their implications for the fulfillment of expectations women have for obstetric care. It is known that previous expectations influence the evaluation of a birth [32]. Thus, the more the expectations of mothers in labor are met, the birth process might be evaluated more positively. In conclusion, the list of seven domains that derived from our study might be used as a checklist of aspects that should be focused on by health professionals in obstetrics to guarantee that the labor and birth experience of women are positive, given the appropriate medical circumstances.

## Conclusions

Based on a sequential mixed-method design that combined literature review with results of focus groups with women and their partners and with the quantitative data of women and professionals, a list of seven domains was derived that considered the key aspects for a mother’s overall experience of labor and birth. This list covers aspects of the caregivers’ competence, support, and communication, the mother’s feelings of safety, fulfilment of her needs, her worries concerning the child’s health and the experienced duration until the first contact with the baby. The results of our study are regarded as a useful contribution to health technology assessment and to research concerning patient-oriented health in the field of obstetrics. In the future, the list of seven domains that was derived from our study might be used as input for developing a birth-specific utility index and a checklist for caregivers in obstetrics to promote a positive labor and birth experience for women, given the medical circumstances.

### Ethical approval

The Medical Ethics Committee of the Leiden University Medical Centre gave approval for this study.

## Abbreviations

QALY: Quality Adjusted Life Year.

## Competing interests

All authors declare that they have no competing interests.

## Authors’ contributions

FG contributed to the conception and design of the study, the acquisition of data, the analysis and interpretation of the data, and drafted the article. LF contributed to the conception and design of the study, the acquisition of data, and interpretation of the data and provided critical revision of the article. MR contributed to the conception and design of the study, the acquisition of data, and interpretation of the data and provided critical revision of the article. JM contributed to the conception and design of the study, interpretation of the data and provided critical revision of the article. KB contributed to the conception and design of the study, interpretation of the data and provided critical revision of the article. AS contributed to the conception and design of the study, interpretation of the data and provided critical revision of the article. EvdA-vM contributed to the conception and design of the study, the acquisition of data, the analysis and interpretation of the data, and provided critical revision of the article. All of the authors provided final approval of the version to be published.

## Pre-publication history

The pre-publication history for this paper can be accessed here:

http://www.biomedcentral.com/1471-2393/14/147/prepub

## Supplementary Material

Additional file 1 Search strategy PubMed, Search strategy Web of Science, Search strategy PsychINFO. Click here for file
