# Supplementary material for: A comprehensive representation of the birth-experience: identification and prioritization of birth-specific domains based on a mixed-method design
Source: BMC Pregnancy Childbirth. 2014 Apr 24;14:147. doi: 10.1186/1471-2393-14-147 (PMC4021407; doi:10.1186/1471-2393-14-147)
Supplement: Additional file 1 — Search strategy PubMed, Search strategy Web of Science, Search strategy PsychINFO. [file 1471-2393-14-147-S1.docx]

**APPENDIX**

**Search strategy PubMed**

("Delivery, Obstetric"[Majr] OR "Parturition"[Majr] OR "Parturition"[ti] OR "childbirth"[ti] OR "Birth"[ti] OR "Labor, Obstetric"[Majr]) AND ("Obstetric Labor Complications"[Majr] OR "Postpartum Hemorrhage"[ti] OR "Parity"[Majr] OR "Pain"[Majr] OR "Pain"[ti] OR "suffering"[ti] OR "Emotions"[Majr] OR "Emotion"[ti] OR "emotions"[ti] OR "fear"[ti] OR "anxiety"[ti] OR "Personal Satisfaction"[Majr] OR "satisfaction"[ti] OR "fulfillment"[ti] OR "Patient Participation"[Majr] OR "involvement"[ti] OR "Self Concept"[Majr] OR "self perception"[ti] OR "self esteem"[ti] OR “control”[ti] OR "Patient Satisfaction"[Majr] OR "Attitude of Health Caregivers"[Majr] OR “assistance”[ti] OR “support”[ti] OR “attitude”[ti] OR "Apgar Score"[Majr] OR "apgar"[ti] OR “umbilical cord pH”[ti]) AND ("Outcome and Process Assessment (Health Care)"[Mesh] OR "Visual Analog Scale"[tiab] OR "VAS"[tiab] OR "questionnaire"[tiab]) NOT "Case Reports" [Publication Type]

**Search strategy Web of Science**

TI=("Delivery" OR "Obstetric" OR "Parturition" OR "childbirth" OR "Labor") AND (TI=("Complications") OR TS=("Postpartum Hemorrhage" OR "Parity" OR "Pain" OR "suffering" OR "Emotions" OR "Emotion" OR "fear" OR "anxiety" OR "satisfaction" OR "fulfillment" OR "Participation" OR "involvement" OR "Self Concept" OR "self perception" OR "self esteem" OR "control" OR (TS=("assistance" OR "support" OR "attitude") AND TS=(midwife OR obstetrician OR professional OR medical)) OR "apgar" OR "umbilical cord pH")) AND (TI=("Outcome") OR TS=("Process Assessment" OR "Visual Analog Scale" OR "VAS" OR "questionnaire"))

**Search strategy PsychINFO**

(DE "Birth" OR DE "Natural Childbirth" OR TI "Parturition" OR TI "childbirth" OR TI "Birth" OR DE "Labor (Childbirth)") AND (DE "Obstetrical Complications" OR TI "Postpartum Hemorrhage" OR DE "Pain Perception" OR DE "Pain" OR TI "Pain" OR TI "suffering" OR DE "Emotional States" OR DE "Anger" OR DE "Anxiety" OR DE "Disappointment" OR DE "Dissatisfaction" OR DE "Distress" OR DE "Doubt" OR DE "Euphoria" OR DE "Fear" OR DE "Frustration" OR DE "Guilt" OR DE "Happiness" OR DE "Suffering" OR TI "Emotion" OR TI "emotions" OR TI "fear" OR TI "anxiety" OR TI "satisfaction" OR TI "fulfillment" OR TI "Participation" OR TI "involvement" OR DE "Self Concept" OR DE "Self Confidence" OR DE "Self Esteem" OR TI "self perception" OR TI "self esteem" OR TI "control" OR DE "Health Caregivers Attitudes" OR TI "assistance" OR TI "support" OR TI "attitude" OR TI "apgar" OR TI "umbilical cord pH") AND (DE "Measurement" OR DE "Questionnaires" OR TX "Visual Analog Scale" OR TX "VAS" OR TX "questionnaire")
